# Supplementary material for: Highly Bright Gold Nanowires Arrays for Sensitive Detection of Urea and Urease
Source: Nanomaterials (Basel). 2022 Nov 16;12(22):4023. doi: 10.3390/nano12224023 (PMC9698401; doi:10.3390/nano12224023)
Supplement: Supplementary file 1 [file nanomaterials-12-04023-s001.zip › nanomaterials-2031905-supplementary.pdf]

Supplementary Materials

Highly Bright Gold Nanowires Arrays for Sensitive Detection of Urea and Urease

Yan Li *, Aowei Zhao, Jieqiong Wang, Jieyu Yu, Fei Xiao and Hongcheng Sun *

College of Material, Chemistry and Chemical Engineering, Key Laboratory of Organosilicon Chemistry and Material Technology, Ministry of Education, Key Laboratory of Organosilicon Material Technology of Zhejiang Province, Hangzhou Normal University, Hangzhou 311121, China
* Correspondence: liyan@hznu.edu.cn (Y.L.); sunhc@hznu.edu.cn (H.S.)

Supporting Figures and Tables

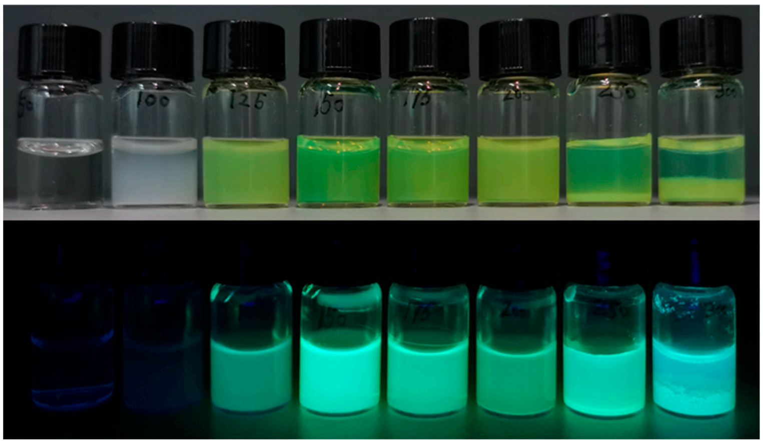

Figure S1 Photograph of Au NWs prepared at different Zn-to-Au ratio.

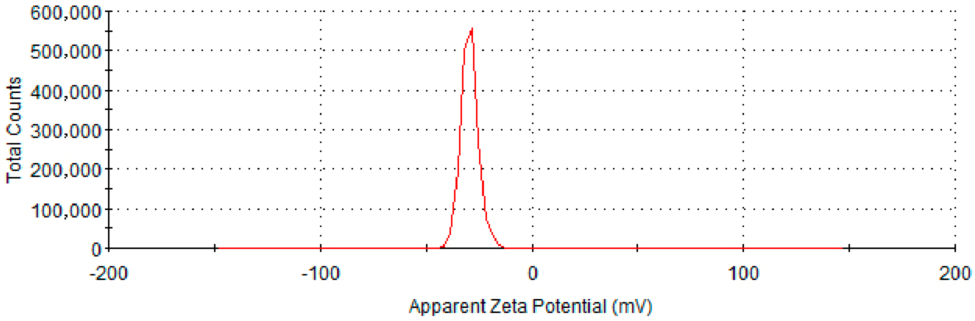

Figure S2 Zeta potential of Au NWs in water.

Table S1 Absolute quantum yield of Au NWs in aqueous solution.

Sample	QY (%)	Mean (%)	RSD (%)
1	32.14		
2	22.54	26.26	5.15

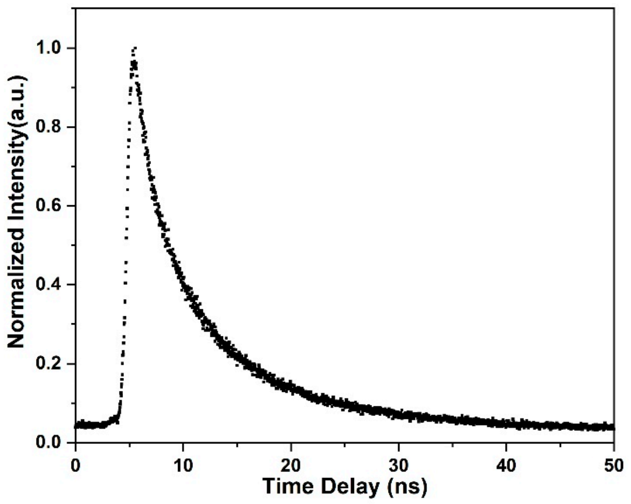

Figure S3 The lifetime of Au NWs.

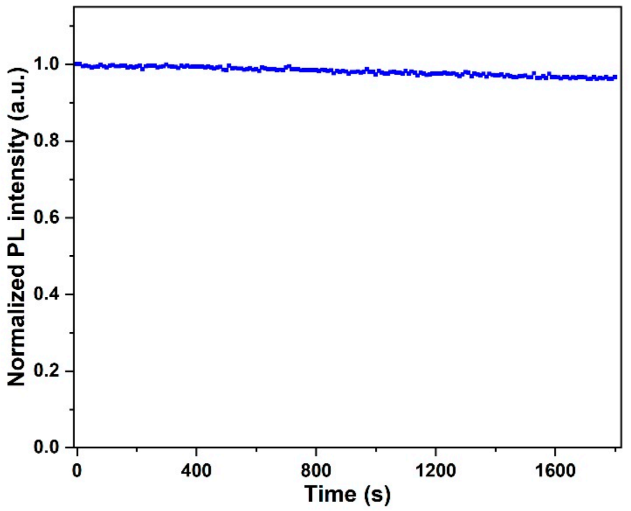

Figure S4 Stability of Au NWs under irradiation.

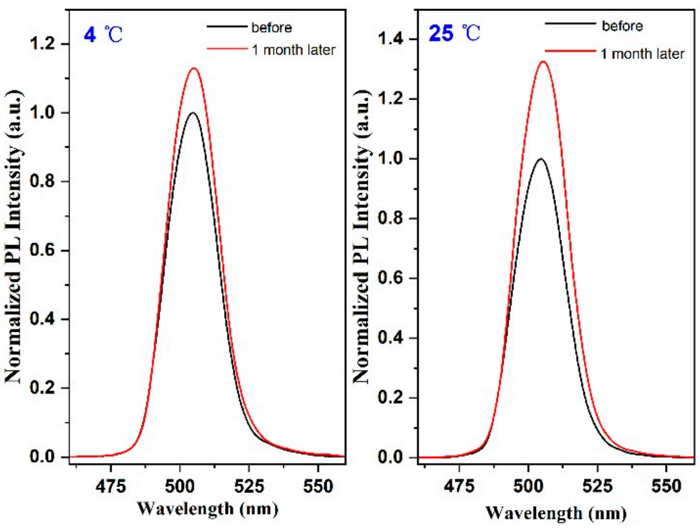

Figure S5 The changes in fluorescence intensity of Au NWs for 1 month at 4 °C and room temperature.

Table S2 Comparison of the proposed method with reported urea-determination methods.

Methods	Materials	pH sensitivity	Range (μM)	LOD (μM)	Ref.
Electrochemistry	Sol-gel	-	2.5-50	2.5	[44]
	Graphite	-	10-250	3	[45]
Colorimetry	Au NPs	6.40-6.60	20-400	5	[46]
	Ag NPs	-	6.25-1000	3.6	[12]
Fluorimetry	MoS_2 QDs	3.8-6.0	5-700	1.8	[47]
	CdSeTe QDs	8.0-11.0	57-13000	10	[47]
	GQDs	7.0-11.0	100-100000	10	[28]
	Au NCs	6.05-6.40	55-550	55	[31]
	Cu NCs	-	250-5000	10	[48]
	Au/Cu NCs	6.0-7.0	5-100	2.23	[29]
This method	Au NWs	7.0-7.8	0-100	2.5	

Table S3 Comparison of the proposed method with reported urease-detection methods.

Methods	Materials	pH sensitivity	Range (U/L)	LOD (u/L)	Ref.
Colorimetry	Au NPs	6.40-6.60	1.8-90	1.8	[46]
	Si QDs	6.0-7.8	2-40	1.07	[11]
Fluorimetry	Si QDs	6.0-7.8	2-40	1.67	[11]
	CMP[a]	4.6-8.6	2-10<br>10-60	0.42	[14]
	$CsPbBr_3$ QDs	4.0-12.0	2.1-550	2.1	[49]
	GQDs	5.0-9.0	50-750	36	[27]

	Au NCs	6.05-6.40	2.2-55	0.55	[31]
This method	Au NWs	7.0-7.8	0-12	0.13	
